# The lymph node ratio as an independent prognostic factor for node-positive triple-negative breast cancer

**DOI:** 10.18632/oncotarget.17413

**Published:** 2017-04-25

**Authors:** Min He, Jia-Xin Zhang, Yi-Zhou Jiang, Ying-Le Chen, Hai-Yuan Yang, Li-Chen Tang, Zhi-Ming Shao, Gen-Hong Di

**Affiliations:** ^1^ Department of Breast Surgery, Key Laboratory of Breast Cancer, Fudan University Shanghai Cancer Center, Shanghai Medical College, Fudan University, Shanghai 200032, China

**Keywords:** lymph node ratio, triple-negative breast cancer, prognosis

## Abstract

**Background:**

We aimed to evaluate the prognostic value of the lymph node ratio (LNR) in patients with axillary lymph node-positive triple-negative breast cancer (TNBC).

**Methods:**

The prognostic efficacy was investigated in the first cohort from the Surveillance, Epidemiology, and End Results (SEER) dataset (n=4114) and was further validated in an independent cohort from Fudan University Shanghai Cancer Center (n=417). Patients were classified into low-, medium- and high-risk LNR groups.

**Results:**

Multivariate analysis revealed that the LNR was an independent predictor of overall survival (hazard ratio (HR) for high-risk LNR: 3.24; 95% confidence interval (CI): 2.56 to 4.09) and breast cancer-specific survival (HR for high-risk LNR: 3.57; 95% CI: 2.76 to 4.62) in the SEER population and also for disease-free survival (HR for high-risk LNR: 4.29; 95% CI: 2.24-8.21) in the validation population. Subgroup analysis revealed that patient classification according to the LNR could discriminate among groups of patients with different survival rates based on pathological nodal (pN) staging.

**Conclusion:**

The LNR shows potential for use as an additional prognostic factor for TNBC patients with positive lymph node involvement. Considering the heterogeneity of TNBC, use of the LNR might allow for optimization of the pN staging system and should be considered when making treatment decisions.

## INTRODUCTION

Triple-negative breast cancer (TNBC) accounts for 10% to 15% of all breast cancers. These tumors lack expression of estrogen receptor (ER) and progesterone receptor (PgR) and gene amplification of human epidermal growth factor receptor 2 (HER2) [1, 2]. TNBC tends to present at a younger age and higher histologic grade with larger tumor size and increased aggressiveness and it has a tendency toward local and visceral metastases rather than bone metastases compared with other types of breast cancer. Compared with patients with other types of breast cancer, those with TNBC experience an early peak of recurrence within the first 3 years and increased mortality within the first 5 years [3, 4].

Among the factors responsible for the poor clinical outcomes of TNBC patients, the local tumor size, regional lymph node status, and distant metastasis are the most important prognostic determinants, and these factors are included in the American Joint Committee on Cancer (AJCC) staging system [5]. The number of involved lymph nodes has great clinical significance in guiding the treatment of breast cancer. A large number of studies have examined nodal status as the most crucial prognostic parameter in TNBC patients [6, 7]. However, other analyses of TNBC patients have shown that their prognoses may not be influenced by the number of positive lymph nodes [8], raising doubt regarding the accuracy and independence of this value in the prediction of clinical outcome. Furthermore, considering the biological heterogeneity among TNBC patients, the current number-based staging system may not provide a reliable estimate of prognosis.

It is well known that the number of involved lymph nodes may be influenced by the number of lymph nodes removed and examined, which in itself depends on the surgical and pathologic procedures performed and thus may be subject to unintended variability. The lymph node ratio (LNR), defined as the number of positive lymph nodes divided by the number removed, standardizes against variability in nodal assessment and has been demonstrated to provide improved prognostic information compared with the number of involved nodes. In a systematic review, Woodward *et al*. [9] have revealed that the LNR is a significant predictor of clinical outcome. Subsequently, Vinh-Hung *et al*. [10] have shown that this ratio is superior to the number of involved nodes as a prognostic indicator. An increasing number of reports in the literature have indicated that the LNR is an independent or an alternative predictor of outcome in node-positive breast cancer patients [11–13]. However, to date, no report has been published regarding its prognostic value in TNBC patients. Furthermore, delineation of a robust and reproducible classification of the LNR that can be applied to identify subgroups of patients with worse outcomes is imperative.

The Surveillance, Epidemiology, and End Results (SEER) Registries began collecting information on HER2 receptor statuses of breast cancer patients in 2010. This article presents the first evaluation of the prognostic value of the LNR in node-positive TNBC using SEER population-based data. We further validated our findings in another independent cohort from Fudan University Shanghai Cancer Center (FUSCC). We aimed to demonstrate that the LNR has potential use for improving the accuracy of prognostic assessments of node-positive TNBC patients.

## RESULTS

### Descriptive statistics of the study population

The basic characteristics of the patients in the two cohorts are presented in Table 1. A total of 4114 TNBC patients from the SEER dataset were included. The median age was 55 years (range of 21 to 75 years). The majority of the patients (64.8%) had at least 10 axillary lymph nodes removed. The median number of involved nodes was 2 (1-76). With regard to the 417 included patients from FUSCC, the median age at diagnosis was 52 years (range of 24 to 79 years). The median numbers of lymph nodes removed and positive lymph nodes were 17 (1-46) and 3 (2-46), respectively. A total of 149 patients (35.7%) received radiotherapy, and most patients (93.0%) received adjuvant chemotherapy after surgery. The median follow-up times for the SEER and FUSCC cohorts were 18 and 51 months, respectively.

**Table 1:** Characteristics of patients with lymph node-positive triple-negative breast cancer from two cohorts

Characteristics	SEER		FUSCC	
	No. of patients (n=4114)	%	No. of patients (n=417)	%
Age, years				
Median	55		52	
Lower-upper quartiles	46–66		44–59	
<50	1361	33.1	172	41.2
≥50	2753	66.9	245	58.8
Race			−	−
White	2929	71.2	−	−
Black	879	21.4	−	−
Other	286	7.0	−	−
Unknown	20	0.5	−	−
Laterality				
Right	2025	49.2	199	47.7
Left	2089	50.8	218	52.3
Tumor size				
0–2cm	1158	28.1	157	37.6
2–5cm	2016	49.0	219	52.5
>5cm	916	22.3	17	4.1
Unknown	24	0.6	24	5.8
Histological grade^a^				
I	28	0.7	0	0.0
II	561	13.6	145	34.8
III	3362	81.7	226	54.2
Unknown	163	4.0	46	11.0
Surgery type				
Mastectomy	2590	63.0	366	87.8
Lumpectomy	1422	34.6	47	11.3
Unknown	102	2.5	4	1.0
No. of lymph nodes removed				
Median	12		17	
Lower-upper quartiles	7–18		14–21	
1-3	566	13.8	3	0.7
4-9	884	21.5	13	3.1
≥10	2664	64.8	401	96.2
No. of positive lymph nodes				
Median	2		3	
Lower-upper quartiles	1-5		1-7	
1-3	2794	67.9	238	57.1
4-9	870	21.1	106	25.4
≥10	451	11.0	73	17.5
Radiotherapy				
Without RT	1693	41.2	259	62.1
With RT	2154	52.4	149	35.7
Unknown	267	6.5	9	2.2
Adjuvant Chemotherapy	−	−		
Without CT	−	−	9	2.2
With CT	−	−	388	93.0
Unknown	−	−	20	4.8

^a^Histological Grade are coded as followings: Well differentiated; Grade I; Moderately differentiated; Grade II; Poorly differentiated; Grade III; Unknown.

### Determination of the prognostic value of the LNR for the SEER dataset

Using the X-tile plots, we classified the patients with an LNR≤0.30 as low risk, those with an LNR of between 0.30 and 0.70 as medium risk, and those with an LNR of over 0.70 as high risk. We analyzed the univariate Kaplan-Meier survival estimates according to risk groups defined by pN staging or defined by the LNR (Supplementary Figure 1). The 3-year OS rates were 77.4%, 66.9%, and 51.0% for the pN1, pN2, and pN3 patients, respectively (P<0.001), while the rates were 81.9%, 65.1% and 46.3% for the patients with low-risk, medium-risk, and high-risk LNRs, respectively (P<0.001). In addition, the 3-year BCSS rates were 80.1%, 70.1% and 54.4% for the pN1, pN2, and pN3 patients, respectively (P<0.001), while the rates were 84.2%, 67.7% and 50.7% for the patients with low-risk, medium-risk, and high-risk LNRs, respectively (P<0.001).

The results of OS and BCSS analyses performed using the Cox proportional hazard regression model are shown in Table 2 and Supplementary Table 1. The significant variables identified in univariate analysis were further analyzed in multivariate analysis. The results revealed that the LNR was an independent and significant prognostic factor for OS and BCSS. Compared with the patients in the low-risk LNR group, the hazard ratios (HRs) of OS were 2.05 (95% confidence intervals [CI], 1.63 to 2.59) for those in the medium-risk group and 3.24 (95% CI, 2.56 to 4.09) for those in the high-risk group. In addition, compared with the patients in the low-risk LNR group, the HRs of BCSS were 2.26 (95% CI, 1.75 to 2.92) for the patients in the medium-risk group and 3.57 (95% CI, 2.76 to 4.62) for those in the high-risk group. Other prognostic factors associated with OS and BCSS included age at diagnosis, tumor histological grade, tumor size, surgery type and receipt of radiation treatment.

**Table 2:** Multivariate analysis of overall survival and breast cancer-specific survival among patients from SEER

Variable	OS		BCSS	
	HR (95% CI)	*P*^c^	HR (95% CI)	*P*^c^
Age at diagnosis	1.02 (1.02–1.03)	**<0.001**	1.01 (1.00–1.02)	**<0.001**
Laterality				
Left-sided	1	−	1	−
Right-sided	1.02 (0.84–1.23)	0.871	1.01 (0.84–1.22)	0.914
Race				
White	1	−	1	−
Black	1.28 (1.02–1.60)	**0.031**	1.18 (0.92–1.51)	0.190
Other^a^	0.67 (0.43–1.06)	0.087	0.63 (0.38–1.04)	0.071
Histological grade^b^				
III	1	−	1	−
I+II	0.69 (0.52–0.92)	**0.011**	0.64 (0.46–0.89)	**0.008**
Tumor size				
<2cm	1	−	1	−
2–5cm	1.59 (1.20–2.10)	**0.001**	1.70 (1.24–2.34)	**0.001**
>5cm	2.95 (2.19–3.40)	**<0.001**	3.17 (2.27–4.44)	**<0.001**
Surgery type				
Mastectomy	1	−	1	−
Lumpectomy	1.36 (1.07–1.73)	**0.012**	1.49 (1.14–1.94)	**0.004**
Radiotherapy				
Without RT	1	−	1	−
With RT	0.56 (0.46–0.68)	**<0.001**	0.56 (0.46–0.70)	**<0.001**
Lymph node ratio				
≤0.30	1	−	1	−
>0.30 and ≤0.7	2.05 (1.63–2.59)	**<0.001**	2.26 (1.75–2.92)	**<0.001**
>0.7	3.24 (2.56–4.09)	**<0.001**	3.57 (2.76–4.62)	**<0.001**

^a^Other includes American Indian/Alaskan native, and Asian/Pacific Islander.

^b^Histological grade are coded as followings: Well differentiated; Grade I; Moderately differentiated; Grade II; Poorly differentiated; Grade III; Unknown.

^c^bold type indicates significance.

### Survival estimates by pN staging stratified by the LNR for the SEER dataset

**Figure 1:**
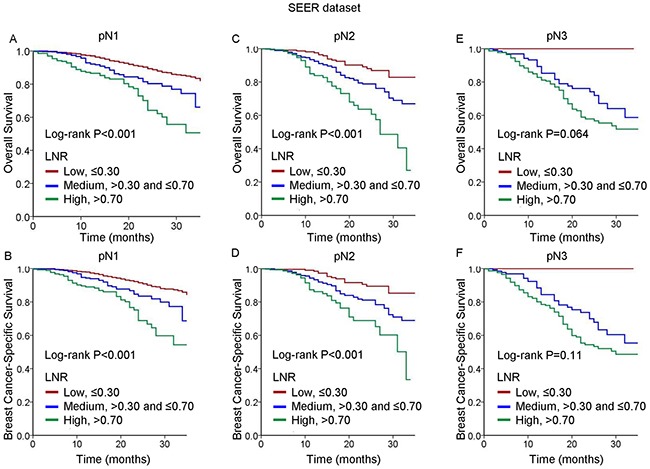
Kaplan-Meier cumulative survival curves generated from the SEER dataset for different lymph node ratios (LNRs) according to different pN stages **(A, C, E)** Overall survival (OS) and **(B, D, F)** breast cancer-specific survival (BCSS) for pN1 **(A, B)**, and pN2 **(C, D)**, and pN3 **(E, F)**.

We further performed subgroup analysis to examine the prognostic value of the LNR in the different pN staging groups. Among all patients, we observed significant differences in OS and BCSS between the patients in the low-risk LNR group and those in the medium- and high-risk LNR groups (P<0.001). We further evaluated the survival rates according to pN staging stratified by the LNR. The results of subgroup analysis of the prognostic significance of the LNR according to the different pN stages are shown in Figure 1. For example, among the pN1 patients, the 3-year OS and BCSS rates were 77.4% and 80.1%, respectively. As the LNR increased, these rates decreased from 81.8% and 84.1%, respectively, for the patients in the low-risk LNR group to 50.6% and 54.3%, respectively, for those in the high-risk LNR group (P<0.001). Similar results were observed for the pN2 patients. Further, When looking at pN3 disease, results showed a trend of differences in OS (P=0.064) and no differences in BCSS (P=0.11) among the three LNR risk groups.

### Validation of the SEER dataset outcomes using the FUSCC dataset

To further validate the findings obtained using the SEER dataset, and especially to assess the prognostic value of the LNR in TNBC, we used data from consecutive patients diagnosed with TNBC between January 2002 and June 2012 at FUSCC. Consistent with the observations in the SEER population, the LNR was found to be a significant prognostic factor for survival. The univariate analysis results for OS and disease-free survival (DFS) are shown in Supplementary Table 2. Based on multivariate analysis, patients in the high-risk group had a significantly worse OS (HR, 3.34; 95% CI 1.56-7.16, P=0.002) and worse DFS (HR, 4.29; 95% CI 2.24-8.21, P<0.001) compared to patients in the low-risk LNR group (Table 3). Further, compared with the patients in the low-risk LNR group, those in the medium-risk group had an increase in the risk of recurrence (HR, 1,88; 95% CI 1.00-3.54, P=0.049) but not in the risk of death (HR, 1,42; 95% CI 0.66-3.08, P=0.37). Radiation treatment and chemotherapy were not associated with OS and DFS in multivariate analysis, which may due to the limited simple size and single-center recruitment.

**Table 3:** Multivariate cox proportional hazard regression model of overall survival and disease-free survival among patients from FUSCC

Variable	OS		DFS	
	HR (95% CI)	*P*^b^	HR (95% CI)	*P*^b^
Age at diagnosis	1.02 (0.99–1.05)	0.268	0.98 (0.96–1.01)	0.209
Laterality				
Left-sided	1	−	1	−
Right-sided	0.95 (0.51–1.78)	0.883	0.71 (0.42–1.20)	0.206
Histological grade^a^				
III	1	−	1	−
II	0.79 (0.40–1.54)	0.481	0.78 (0.50–1.35)	0.371
Tumor size				
<2cm	1	−	1	−
2–5cm	1.82 (0.88–3.77)	0.109	2.53 (1.35–4.74)	**0.004**
>5cm	3.13 (0.80–12.22)	0.100	4.62 (1.56–13.68)	**0.006**
Surgery type				
Mastectomy	1	−	1	−
Lumpectomy	0.000 (0.000-)	0.975	0.57 (0.17–1.91)	0.365
Radiotherapy				
Without RT	1	−	1	−
With RT	0.80 (0.39–1.65)	0.804	0.81 (0.46–1.43)	0.474
Chemotherapy				
Without CT	1	−	1	−
With CT	1.13 (0.23–5.55)	0.882	1.98 (0.25–15.72)	0.517
Lymph node ratio				
≤0.30	1	−	1	−
>0.30 and ≤0.7	1.42 (0.66–3.08)	0.370	1.88 (1.00–3.54)	**0.049**
>0.7	3.34 (1.56–7.16)	**0.002**	4.29 (2.24–8.21)	**<0.001**

^a^Histological grade are coded as followings: Well differentiated; Grade I; Moderately differentiated; Grade II; Poorly differentiated; Grade III; Unknown.

^b^bold type indicates significance.

**Figure 2:**
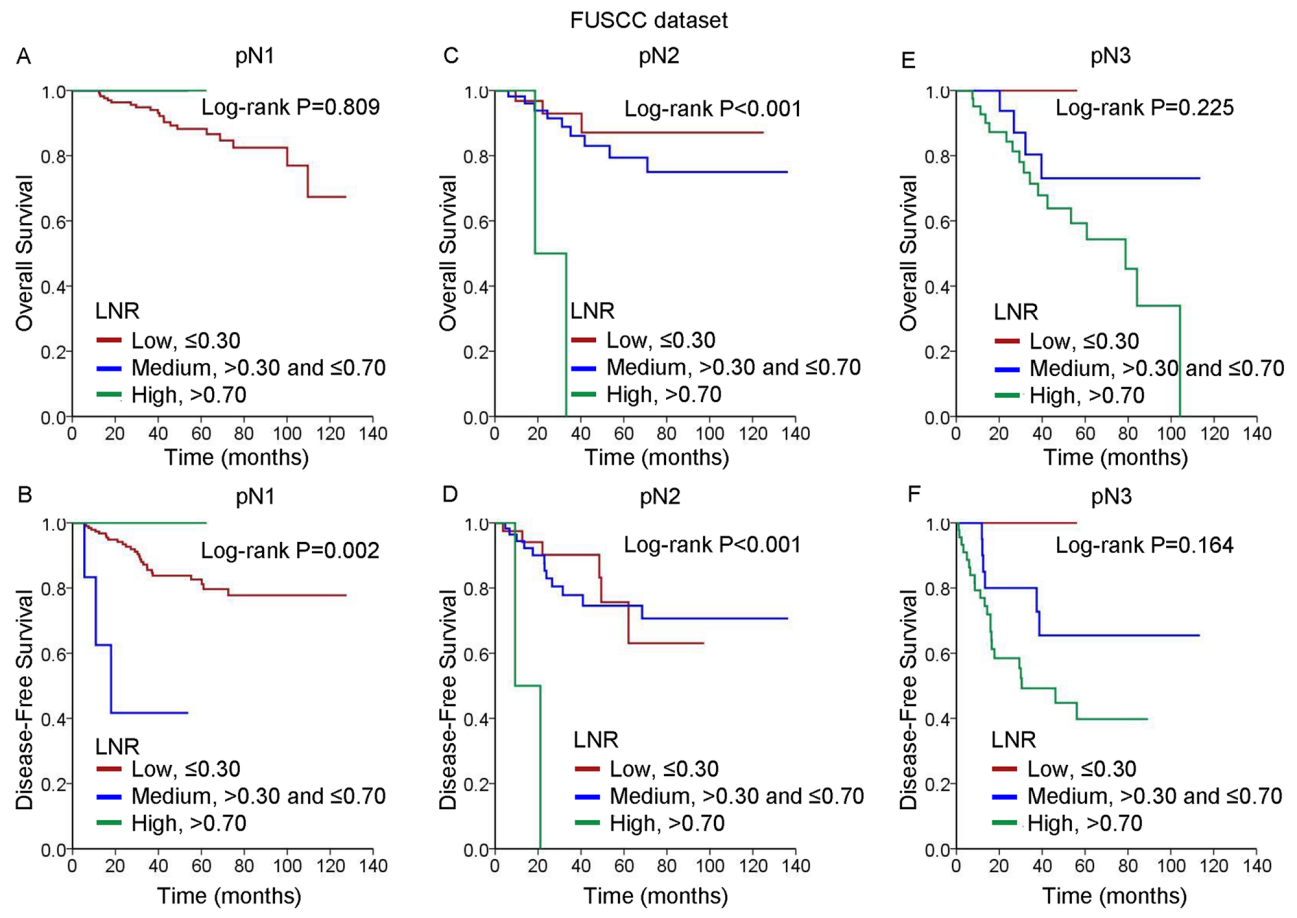
Kaplan-Meier cumulative survival curves generated from the FUSCC dataset for different lymph node ratios (LNRs) according to different pN stages **(A, C, E)** Overall survival (OS) and **(B, D, F)** disease-free survival (DFS) for pN1 **(A, B)**, and pN2 **(C, D)**, and pN3 **(E, F)**.

We further analyzed the univariate Kaplan-Meier survival estimates according to risk groups defined by pN staging or defined by the LNR (Supplementary Figure 2). The Kaplan-Meier estimates of OS and DFS for each of the three pN staging subgroups stratified by the LNR are shown in Figure 2. Among the pN2 and pN3 patients, consistent with the observations in the SEER population, a higher LNR was associated with increases in the risks of death and recurrence. However, among the pN1 patients, no differences in OS were detected among the LNR risk groups. In addition, the trends in DFS differed between the pN2 and pN3 patients, and the patients with a medium-risk LNR experienced poorer DFS compared with those with a high-risk LNR.

## DISCUSSION

In this study, we sought to evaluate the prognostic value of the LNR in two cohorts of patients with node-positive TNBC. Using a large population cohort from the SEER dataset, we proposed an LNR classification model that was used to classify the patients into low-risk, medium-risk and high-risk groups according to their different LNRs. After making adjustments for other prognostic factors, we found that the LNR provided additional prognostic risk information based on the traditional pN staging classification system among the patients with lymph node-positive TNBC. Despite the ethnic heterogeneity, the prognostic effect of LNR was subsequently successfully validated in another independent cohort from FUSCC. Our findings indicated that the LNR was a prognostic factor for survival in TNBC patients with positive lymph node involvement.

Despite advances in sentinel node biopsy techniques, adequate dissection of axillary lymph nodes remains the most important method for accurate disease staging and has subsequent therapeutic implications in patients with clinically lymph node-positive breast cancer. Although there is no recommended minimum number of nodes that should be removed in axillary lymph node dissection, it is generally accepted that a minimum of 10 axillary nodes are required for adequate staging [17]. However, due to variations in the procedures for lymph node clearance and differences in physical examination findings, heterogeneity among results of lymph node examinations is commonly encountered in daily practice. To address this heterogeneity and to improve the comparison between centers, one should intuitively take not only the number of positive lymph nodes but also the number of nodes examined into account. Compared with the number-based staging system, the LNR classification model exploits additional information on the total number of lymph nodes removed. Increasing evidence is establishing the prognostic role of the LNR in breast cancer [10, 18–21]. Our results support previous findings, demonstrating that the LNR is a prognostic factor in both multivariate and univariate analyses. The consistency of the results obtained from the two independent populations reinforced the prognostic value of this ratio. Subsequently, we categorized the patients with different pN stages according to their LNRs, assigning them to low-, medium-, and high-risk LNR groups, respectively. As expected, there were significant differences among the Kaplan-Meier survival estimates for the three LNR groups (Figures 1 and 2). We demonstrated these patients with different pN stages could be further classified into heterogeneous prognostic subsets according to the LNR, thereby improving the prognostication system for patients with node-positive TNBC.

The importance of the LNR has been addressed by many investigators; however, the cutoff value for this ratio has varied widely among studies [22–24], resulting in difficulty in establishing staging criterion. Using a bootstrap sampling method, Vinh-Hung *et al*. [10] have reported LNR cutoff values for classification of patients into low- (≤0.20), medium- (0.21-0.65), and high-risk (>0.65) LNR groups. They have proposed that LNR cutoff values predict breast cancer prognosis more accurately than pN categories and that this method of classification could be considered as an alternative to pN staging. However, there is limited information regarding the prognostic value of the LNR in TNBC specifically. With inclusion of a large number of TNBC cases from the SEER dataset in our study, we were able to classify all node-positive patients into three LNR categories using the X-tile plots method, which does not require any predefined assumptions or distributional specifications. Our method of LNR categorization showed a clear advantage over traditional pN staging. In the SEER dataset, the HR of OS significantly differed between the high- and low-risk LNR groups (HR=3.24), and this difference was greater than that between pN3 and pN1 (HR=2.72). Moreover, the HR of BCSS of 3.57 for the high-risk LNR group demonstrated a separation between the high- and low-risk groups (HR=3.57) that was wider than that between pN3 and pN1 (HR=2.97) (Supplementary Table 3). In addition, among the pN1 and pN2 patients categorized by traditional pN staging, classification by the LNR could further distinguish among patients with differing mortality risks. In contrast, among the pN1 cohort from FUSCC, the patients in the medium-risk LNR group experienced poorer OS and DFS compared with those in the high-risk LNR group, which was probably due to the small sample size.

For decades, the absolute number of positive axillary lymph nodes has traditionally been accepted as an important prognostic factor in breast cancer patients. However, Hernandez-Aya *et al*. [8] have proposed that distinct from other subtypes of breast tumors in which the number of positive lymph nodes is correlated with prognosis, OS and DFS estimates are not greatly influenced by the number of additional positive lymph nodes in TNBC patients. In contrast, our study demonstrated that the extent of positive lymph nodes remained an independent prognostic factor in TNBC patients with lymph node involvement (Supplementary Table 3). These results are in accordance with previous studies showing that the absolute number of positive lymph nodes affects the prognosis of TNBC patients [25–27].

To appreciate our findings, some strengths and limitations should be mentioned. To the best of our knowledge, this is the largest study evaluating the prognostic significance of the LNR in patients with node-positive TNBC. The sizable number of TNBC patients in the SEER dataset that were assessed supports the validity and objectivity of our conclusions. In addition, we verified our results from the SEER dataset in an independent cohort, demonstrating consistent conclusions. Inevitably, our study has several limitations. First, the SEER dataset lacks several important variables, such as adjuvant chemotherapy and recurrence types. We could not adjust for additional confounding factors. Second, information regarding HER-2 status was not available in the SEER dataset until 2010; thus, we assessed short-term survival status after initial diagnosis. This limitation was partially compensated for by validation using the FUSCC dataset, which had a median follow-up of 51 months. Third, our study was performed using two retrospective datasets rather than prospective cohorts; this approach might have introduced unaccounted sampling biases. Lastly, patients with 1 to 3 lymph nodes removed account for 13.8% in the SEER dataset, which has to be considered as a confounding factor.

In conclusion, we have shown that in two independent cohorts of TNBC patients with positive lymph node involvement, the LNR appears to serve as an additional prognostic factor based on traditional pN staging. Considering the heterogeneity and aggressive behavior of TNBC, further studies are needed to verify this prognostic factor and to develop standard classifications that accurately reflect the clinical behavior of this disease, thereby guiding treatment approaches.

## PATIENTS AND METHODS

We collected information on female breast cancer patients treated between January 1, 2010 and December 31, 2012 from the SEER dataset. Patients diagnosed with breast cancer before 2010 were excluded from this study because of unavailable HER2 data, and 4114 patients were included who met the following criteria: female, age of diagnosis of between 20 and 75 years, breast cancer as the primary and only cancer diagnosis, unilateral breast cancer, pathologically confirmed infiltrating ductal or lobular carcinoma, subtype of TNBC, one or more involved lymph nodes, known tumor size, histological grades I to III and AJCC stages I to III.

The primary study outcomes of the SEER data were OS and BCSS. OS was defined as the time from the date of diagnosis to the date of death due to all causes (including breast cancer) or to the date of last follow-up. BCSS was calculated from the date of diagnosis to the date of breast cancer death. Patients who died of other causes were censored at the date of death.

To validate the preliminary findings obtained from the SEER database, we used data from 417 consecutive patients diagnosed with AJCC stage Ito IIIC unilateral TNBC who were treated between January 2002 and June 2012 at FUSCC. All included cases were histopathologically re-confirmed independently by two experienced pathologists according to the ASCO/CAP 2010 criteria. The cutoff for ER or PgR positivity was ≥ 1% of tumor cells with nuclear staining [14]. Cytoplasmic staining was ignored [15]. Pathologic HER2 status was defined according to the ASCO/CAP guidelines [16]. Patients were excluded if they received neoadjuvant chemotherapy or had pathologically node negative disease. Other specific inclusion criteria were as follows: female sex, between 20 and 79 years of age at diagnosis, unilateral breast cancer with documented primary site and exclusive laterality, pathologically confirmed invasive breast carcinoma, and known tumor size, as well as positive lymph node status.

For the FUSCC dataset, the outcomes of interest were OS and DFS. DFS was calculated from the date of diagnosis to the date of first event of local, regional, or distant metastasis of breast cancer. Our study was approved by an independent ethics committee/institutional review board at FUSCC (Shanghai Cancer Center Ethical Committee). All patients provided written informed consent.

### Statistical analyses

Using X-tile plots (X-tile software version 3.6.1, Yale University School of Medicine, New Haven, CT, USA), the patients were categorized according to their LNR into one of three categories: low risk (LNR ≤0.30), medium risk (LNR >0.30 and ≤0.70) and high risk (LNR >0.70). Based on the pathology review, the number of positive lymph nodes was categorized into one of three groups: pN1 (one to three positive lymph nodes), pN2 (four to nine positive lymph nodes), and pN3 (≥10 positive lymph nodes).

Adjusted HRs along with 95% CIs were calculated using the Cox proportional hazards regression model. Survival curves were estimated using the Kaplan-Meier method, and the log-rank test was used to test for differences between groups. Survival time was estimated using the life-table method. Two-sided P values of less than 0.05 were considered statistically significant. All statistical analyses were carried out using the SPSS version 20.0 software package (IBM SPSS Statistics, Chicago, IL, US).

## SUPPLEMENTARY MATERIALS FIGURES AND TABLES



## Abbreviations

LNR: lymph node ratio
TNBC: triple-negative breast cancer
SEER: Surveillance, Epidemiology, and End Results
HR: hazard ratio
CI: confidence interval
pN: pathological nodal
ER: estrogen receptor
PgR: progesterone receptor
HER2: human epidermal growth factor receptor 2
AJCC: American Joint Committee on Cancer
FUSCC: Fudan University Shanghai Cancer Center
OS: overall survival
BCSS: breast cancer-specific survival
DFS: disease-free survival
CAP: College of American Pathologists
