# Supplementary material for: The lymph node ratio as an independent prognostic factor for node-positive triple-negative breast cancer
Source: Oncotarget. 2017 Apr 25;8(27):44870–80. doi: 10.18632/oncotarget.17413 (PMC5546527; doi:10.18632/oncotarget.17413)
Supplement: Supplementary file 1 [file oncotarget-08-44870-s001.pdf]

The lymph node ratio as an independent prognostic factor for node-positive triple-negative breast cancer

SUPPLEMENTARY FIGURES AND TABLES

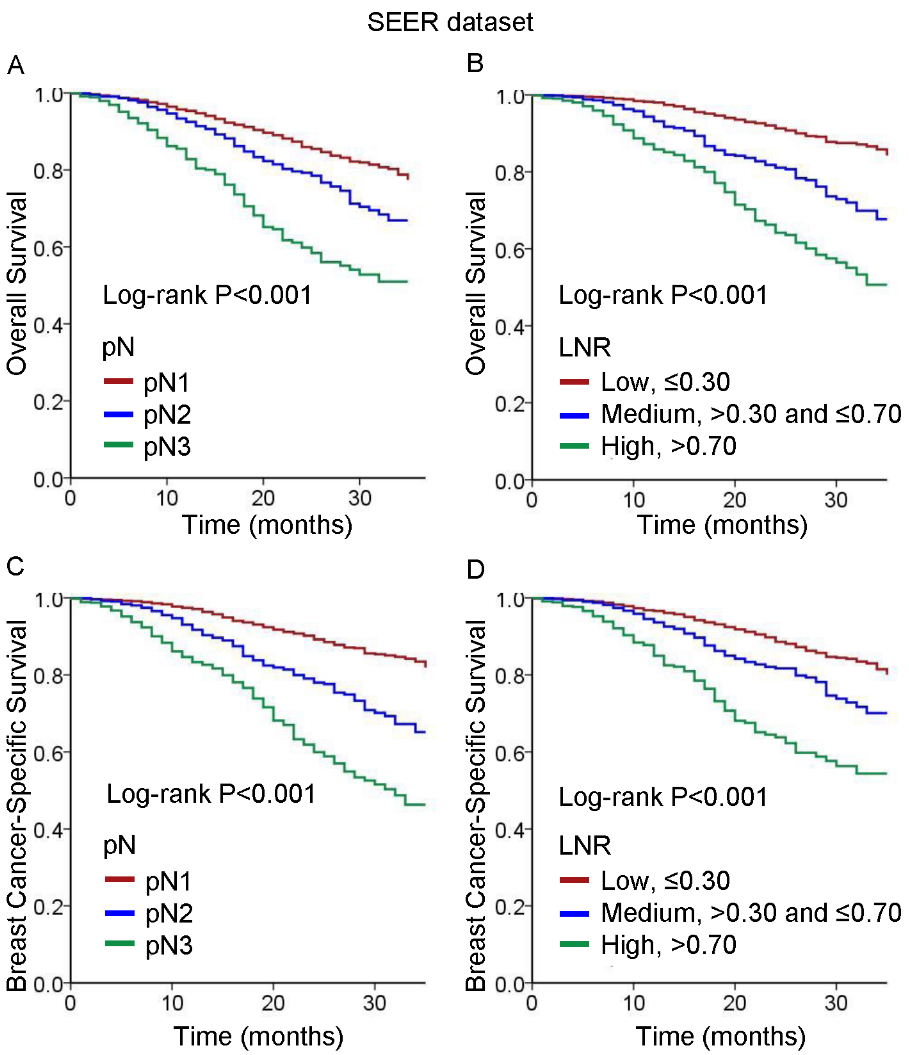

Supplementary Figure 1: Kaplan-Meier cumulative survival curves generated from the SEER dataset for different pN stages and lymph node ratios (LNRs). **(A, B)** Overall survival (OS) and **(C, D)** breast cancer-specific survival (BCSS) for pN **(A, C)** and the LNR **(B, D)**.

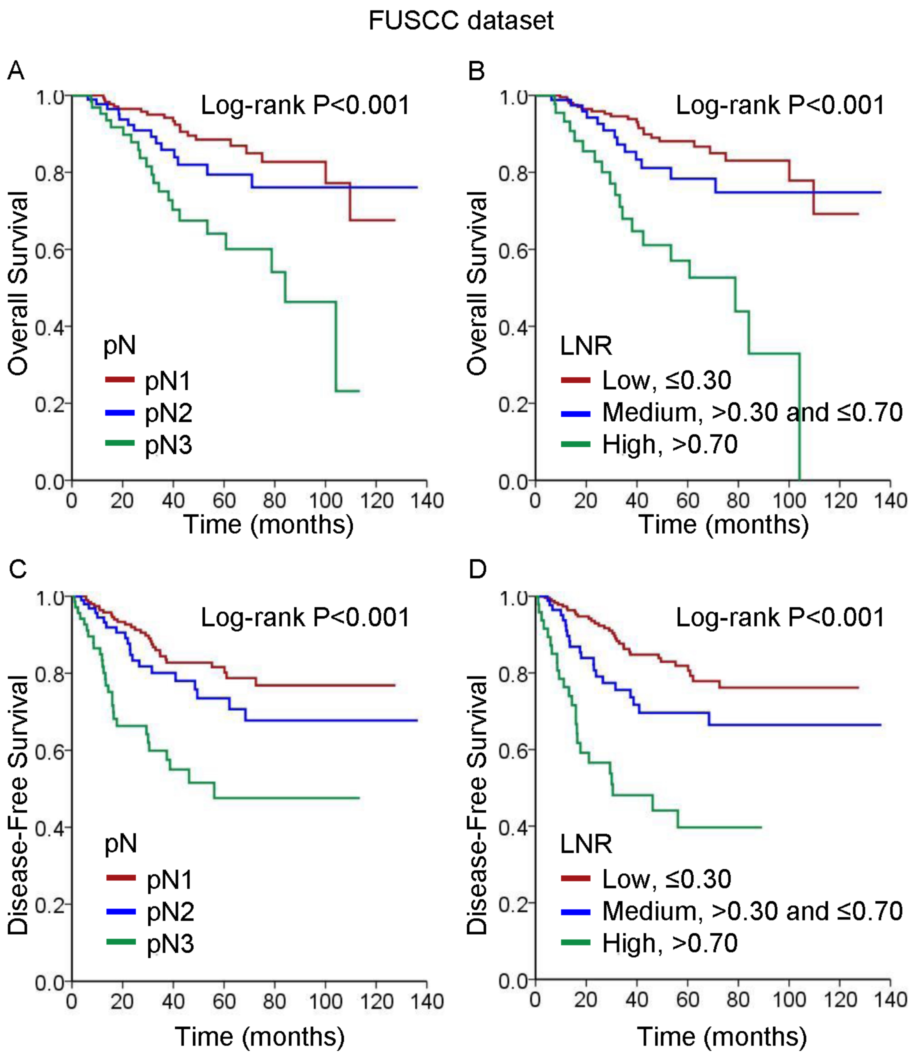

Supplementary Figure 2: Kaplan-Meier cumulative survival curves generated from the FUSCC dataset for different pN stages and lymph node ratios (LNRs). **(A, B)** Overall survival (OS) and **(C, D)** disease-free survival (DFS) for pN **(A, C)** and the LNR **(B, D)**.

Supplementary Table 1: Univariate analysis of overall survival and breast cancer-specific survival among patients from SEER

	OS		BCSS	
**Variable**	**HR (95% CI)**	***P*[c]**	**HR (95% CI)**	***P*[c]**
Age at diagnosis, continuous	1.03 (1.02-1.03)	**<0.001**	1.02 (1.01-1.02)	**<0.001**
Laterality				
Left-sided	1	-	1	-
Right-sided	1.05 (0.88-1.25)	0.617	1.03 (0.85-1.25)	0.739
Race				
White	1	-	1	-
Black	1.30 (0.88-1.94)	0.193	1.37 (0.88-2.13)	0.167
Other[a]	1.62 (1.06-2.46)	**0.026**	1.62 (1.01-2.58)	**0.045**
Histological grade[b]				
III	1	-	1	-
I+II	0.80 (0.61-1.05)	0.105	0.71 (0.52-0.96)	**0.029**
Tumor size				
<2cm	1	-	1	-
2-5cm	1.59 (1.23-2.07)	**<0.001**	1.70 (1.27-2.29)	**<0.001**
>5cm	4.19 (3.23-5.43)	**<0.001**	4.83 (3.60-6.47)	**<0.001**
Surgery type				
Mastectomy	1	-	1	-
Lumpectomy	2.07 (1.67-2.56)	**<0.001**	0.43 (0.34-0.55)	**<0.001**
Radiotherapy				
Without RT	1	-	1	-
With RT	0.55 (0.46-0.66)	**<0.001**	0.58 (0.48-0.71)	**<0.001**
Lymph node ratio				
≤0.30	1	-	1	-
>0.30 and ≤0.7	2.27 (1.83-2.83)	**<0.001**	2.48 (1.95-3.15)	**<0.001**
>0.7	4.50 (3.66-5.54)	**<0.001**	4.83 (3.84-6.08)	**<0.001**

[a]Other includes American Indian/Alaskan native, and Asian/Pacific Islander.
[b]Histological grade are coded as followings: Well differentiated; GradeI; Moderately differentiated; Grade II; Poorly differentiated; Grade III; Unknown.
[c]bold type indicates significance

Supplementary Table 2: Univariate analysis of overall survival and disease-free survival among patients from FUSCC

	OS		DFS	
**Variable**	**HR (95% CI)**	***P*[b]**	**HR (95% CI)**	***P*[b]**
Age at diagnosis	1.03 (1.01-1.06)	**0.006**	1.01 (0.99-1.03)	0.495
Laterity				
Left-sided	1	-	1	-
Right-sided	0.94 (0.55-1.60)	0.814	0.80(0.51-1.25)	0.320
Histological grade[a]				
III	1	-	1	-
II	0.73 (0.41-1.30)	0.282	0.73(0.45-1.20)	0.216
Tumor size				
<2cm	1	-		
2-5cm	1.82 (0.93-3.54)	0.081	2.16(1.24-3.75)	**0.006**
>5cm	5.59 (2.10-14.93)	**0.001**	4.44(1.74-11.28)	**0.002**
Surgery type				
Mastectomy	1	-	1	-
Lumpectomy	0.34(0.08-1.38)	0.129	0.43(0.16-1.17)	0.097
Radiotherapy				
Without RT	1	-	1	-
With RT	0.80(0.44-1.47)	0.799	0.97(0.60-1.56)	0.895
Adjuvant Chemotherapy				
Without CT	1	-	1	-
With CT	1.75(0.54-5.66)	0.347	1.11(0.27-4.55)	0.88
Lymph node ratio				
≤0.30	1	-	1	-
>0.30 and ≤0.7	1.47(0.74-2.91)	0.270	1.89(1.09-3.26)	**0.023**
>0.7	4.89(2.65-8.99)	**<0.001**	4.89(2.89-8.28)	**<0.001**

[a]Histological grade are coded as followings: Well differentiated; Grade I; Moderately differentiated; Grade II; Poorly differentiated; Grade III; Unknown.
[b]bold type indicates significance.

Supplementary Table 3: Effect of lymph node ratio and pN classification on survival among patients from SEER

	OS		BCSS	
**Variable**	**HR[a] (95% CI)**	***P*[b]**	**HR[a] (95% CI)**	***P*[b]**
Lymph node ratio				
Low,≤0.30	1	-	1	-
Medium, >0.30 and ≤0.70	2.05 (1.63-2.59)	**<0.001**	2.26 (1.75-2.92)	**<0.001**
High,>0.70	3.24 (2.56-4.09)	**<0.001**	3.57 (2.76-4.62)	**<0.001**
pN				
N1	1	-	1	-
N2	1.53 (1.21-1.93)	**<0.001**	1.63 (1.27-2.10)	**0.001**
N3	2.72 (2.14-3.46)	**<0.001**	2.97 (2.29-3.85)	**<0.001**

[a]Cox proportional hazards model. Hazard ratios are adjusted for age, race, histologic grade, tumor size, surgery type and radiotherapy.
[b]bold type indicates significance.
